# Sunday Games

**Published:** 1921-02-26

**Authors:** 


### Sunday Games.

### SABBATARIANISM OR GOOD SENSE?

It is to be hoped that the Ministry of Health will take an early opportunity of framing some definite policy on the question of Sunday games. It is a matter of considerable importance, for it directly affects the health of the younger generation, and there is plenty of evidence to show that the spirit of narrow Sabbatarianism still effectively operates in local authorities when proposals for giving facilities for Sunday recreation are put forward. The latest illustration of this is provided in the refusal of the London County Council to allow Sunday games to be played in the parks and commons under their control. The very remarkable excuse advanced is that there is no demand for such a privilege. We should like to know on what grounds the Parks Committee of the Council base that assumption. There are in London many thousands of young people who would eagerly welcome the opportunity of playing football, cricket, tennis, hockey, or other games on Sunday, and who are the worse off in more ways than one through lack of it. If the Council is in any doubt upon the point it could very soon be convinced by taking a plebiscite of the various young men's and young women's organisations in its areas. Or, better still, it might easily test the point by throwing open a few playing-grounds situated widely apart, and observing the result. Apart from ancient prejudice, there is really no case against the playing of open-air games on Sunday. Scarcely a document issued from Dr. Addison's Department dealing with the health of the people or a report of any kind sent out by the Board of Education fails to lay stress on the importance of encouraging open-air sports among the young. All expert opinion is agreed that one of the most powerful and essential retorts to modern industrialism and all that it implies is the provision of local and national playgrounds easy of access for all and the fostering of a youthful enthusiasm for healthful exercise. If we are honest we must surely agree that there is no better day than Sunday on which to make these facilities general. Sunday games are not only calculated to lay the foundations of health and improve the physique of the population at the critical age; they will also, as was properly pointed out to the London County Council a few days ago, do away with "the loafing, the street-corner gambling, and all the evils of enforced idleness," and thus in a very real way accomplish the very purpose implied in the protest of old-fashioned protectors of the Sabbath. We feel confident that if this matter is brought directly to the notice of the Minister of Health it will not be difficult to induce him to bring official pressure to bear upon the County Council or any other loc[illegible] authorities that take an equally short-sighted view.

